# GATA2-driven ZNF486-JAM2 activation collaboratively counteracts epithelial-mesenchymal transition and *in vitro* invasion in endometrial cancer

**DOI:** 10.3389/fonc.2026.1931641

**Published:** 2026-09-15

**Authors:** Xing Fan, Zhengjiao Tong, Yingxia Ou, Xiaoli Wang, Rong Wang

**Affiliations:** 1 Department of Obstetrics & Gynecology, Changsha Hospital for Maternal & Child Health Care Affiliated to Hunan Normal University, Changsha, China; 2 Department of Medical, Hunan Normal University, Changsha, China

**Keywords:** epithelial-mesenchymal transition, GATA2, invasion, Jam2, migration, transcriptional regulation, uterine corpus endometrial carcinoma, ZNF486

## Abstract

**Objective:**

To investigate the role and molecular mechanism of the GATA2/ZNF486/JAM2 transcriptional regulatory axis in the invasion and migration of Uterine Corpus Endometrial Carcinoma (UCEC).

**Methods:**

We employed reverse transcription quantitative polymerase chain reaction (RT-qPCR) and Western blot (WB) to assess the expression levels of ZNF486, GATA2, and JAM2 in UCEC cell lines. Lentivirus-mediated overexpression and knockdown cell lines were established, and CCK-8, colony formation, Transwell, and wound healing assays were performed to evaluate cell proliferation, invasion, and migration. WB and immunofluorescence were used to detect changes in epithelial-mesenchymal transition (EMT) markers. The direct binding and transcriptional activation of target gene promoters by transcription factors were confirmed through ChIP-qPCR and dual-luciferase reporter assays.

**Results:**

ZNF486 was significantly downregulated in UCEC. Its overexpression upregulated E-cadherin, downregulated N-cadherin and Vimentin, and markedly inhibited cell proliferation, invasion, and migration. Mechanistically, ZNF486 directly bound to and transcriptionally activated the JAM2 promoter; knockdown of JAM2 partially attenuated the tumor-suppressive effects of ZNF486. Furthermore, GATA2 was identified as an upstream transcription factor that directly activated ZNF486 transcription; knockdown of ZNF486 abrogated the tumor-suppressive effects induced by GATA2 overexpression.

**Conclusion:**

GATA2 inhibits EMT, invasion, and migration in UCEC by transcriptionally activating the ZNF486-JAM2 axis. This pathway provides a potential molecular framework for understanding UCEC progression.

## 1 Introduction

Uterine Corpus Endometrial Carcinoma (UCEC) is one of the most common malignancies of the female reproductive system, with its incidence continuing to rise globally (1, 2). Although patients with early-stage UCEC have a favorable prognosis, a subset still experience recurrence or metastasis, leading to poor survival outcomes (3). Tumor invasion and metastasis are critical processes contributing to treatment failure and poor prognosis, involving complex biological events such as epithelial-mesenchymal transition (EMT), cytoskeletal remodeling, and extracellular matrix degradation (4–6). Therefore, a deeper understanding of the molecular regulatory networks governing UCEC invasion and metastasis is essential for identifying novel diagnostic biomarkers and therapeutic targets.

Zinc finger protein 486 (ZNF486) belongs to the Krüppel-type zinc finger transcription factor family, whose members are widely involved in regulating cell proliferation, differentiation, apoptosis, and tumorigenesis (7, 8). ZNF486, like many family members, contains an N-terminal KRAB domain and is typically associated with transcriptional repression. Increasing evidence has revealed the aberrant expression and dysfunction of zinc finger proteins in various malignancies (9). Our recent study, based on cervical exfoliated cell samples, demonstrated that ZNF486 methylation levels differ significantly between UCEC patients and benign controls (*P* = 0.0051), suggesting that ZNF486 may be silenced by promoter hypermethylation in UCEC (10). However, that study focused primarily on the diagnostic value of ZNF486 methylation, leaving its biological functions and regulatory networks in UCEC pathogenesis largely unexplored.

Junctional adhesion molecule 2 (JAM2), a member of the immunoglobulin superfamily, is primarily localized at tight junctions of epithelial and endothelial cells, where it participates in maintaining cell polarity, regulating barrier function, and modulating leukocyte migration (11). JAM2 has been identified as a core hub gene commonly downregulated in both endometrial and ovarian cancers through bioinformatics analysis (12), suggesting a potential tumor-suppressive role for JAM2 in UCEC. However, the expression regulation and biological functions of JAM2 in UCEC remain largely unknown. Furthermore, although JAM2’s tumor-suppressive functions have been documented in lung, breast, and colorectal cancers (13–15), its upstream transcriptional regulators in UCEC remained undefined. Given the bioinformatic correlation between ZNF486 and JAM2, we selected JAM2 as a candidate downstream target for functional validation.

GATA2, a critical member of the GATA transcription factor family, plays an essential role in hematopoietic stem cell self-renewal and differentiation (16). In UCEC, Liao et al. reported that GATA2 is downregulated and that melatonin suppresses UCEC cell proliferation and metastasis by upregulating GATA2 (17). Suhail et al. demonstrated through CRISPR-Cas9 knockout that GATA2 is a key transcription factor regulating the invasiveness of endometrial stromal cells (18). Collectively, these findings underscore the important regulatory functions of GATA2 in endometrial diseases. Nevertheless, Nevertheless, although studies like that by Polaki et al. have identified SIN3B as a downstream target of GATA2 in uterine serous carcinoma (19), the broader downstream target gene network of GATA2 in UCEC, including its potential regulatory relationship with ZNF486, remains largely unexplored.

To address these knowledge gaps, we hypothesized that GATA2, ZNF486, and JAM2 form a novel transcriptional regulatory axis in UCEC. Specifically, we propose that GATA2 acts as an upstream activator of ZNF486, which in turn transcriptionally regulates JAM2. The present study aims to systematically investigate the role of this GATA2/ZNF486/JAM2 axis in UCEC invasion and migration, and to elucidate whether its function is mediated through the regulation of EMT-associated molecular changes.

## 2 Methods

### 2.1 Cell culture

The human UCEC cell lines Ishikawa and HEC-1A, as well as the immortalized normal endometrial epithelial cell line EM-E6/E7/TERT-1, were obtained from commercial sources (**Supplementary Table S1**). All cells were cultured in DMEM/F12 (Dulbecco’s Modified Eagle Medium/Nutrient Mixture F-12) medium (Gibco, USA) supplemented with 10% fetal bovine serum (FBS, Gibco, USA), 100 U/mL penicillin, and 100 μg/mL streptomycin (Gibco, USA) at 37 C in a humidified atmosphere containing 5% CO_2_. Cells were routinely passaged according to standard procedures.

### 2.2 Lentiviral construction and infection

Lentiviruses encoding ZNF486 overexpression (oe-ZNF486), GATA2 overexpression (oe-GATA2), and their corresponding empty vector controls (oe-NC), as well as short hairpin RNAs targeting ZNF486 (sh-ZNF486), JAM2 (sh-JAM2), and negative control (sh-NC), were constructed and packaged by a commercial vendor (GeneChem, China). For lentiviral infection, cells were incubated with lentiviral particles in the presence of 8 μg/mL polybrene (Sigma-Aldrich, USA). Following infection, stably transduced cell lines were selected with appropriate antibiotics (puromycin or blasticidin) for 7–10 days. Overexpression or knockdown efficiencies were validated by WB and/or reverse transcription quantitative polymerase chain reaction (RT-qPCR).

### 2.3 Bioinformatic analysis

The GEPIA2 database (http://gepia2.cancer-pku.cn/) was utilized to analyze the mRNA expression differences of GATA2, ZNF486, and JAM2 between tumor and normal tissues, as well as their pairwise expression correlations, based on the TCGA-UCEC dataset. The cBioPortal platform (https://www.cbioportal.org/) was further employed to validate the co-expression correlation between ZNF486 and JAM2. The JASPAR database (https://jaspar.genereg.net/) was used to predict potential binding motifs of ZNF486 in the JAM2 promoter region and GATA2 binding sites in the ZNF486 promoter region. The JASPAR 2024 database was accessed on December 18, 2025. A relative profile score threshold of 80% was used to identify potential binding sites. Additionally, the TF-Target Finder tool (https://ccg.epfl.ch//tf_target_finder/) was applied to predict upstream transcription factors of ZNF486. GEPIA2 analysis was performed using the TCGA-UCEC and GTEx datasets in January 2026. Correlation analysis was performed using the Spearman method.

### 2.4 RNA extraction and RT-qPCR

Total RNA was extracted from cells using TRIzol reagent (Invitrogen, USA) according to the manufacturer’s instructions. Briefly, 1 μg of total RNA was reverse-transcribed into cDNA using the PrimeScript™ RT Reagent Kit (Takara, Japan). The resulting cDNA was then amplified using SYBR Green Master Mix (Bio-Rad, USA) on a CFX96 Real-Time PCR Detection System (Bio-Rad, USA). β-actin served as the internal control, and relative gene expression was calculated using the 2^-^ΔΔCt method. Primer sequences are listed in **Supplementary Table S2**. β-actin was used as a reference gene as its expression was stable across all cell lines and treatments in our pilot experiments.

### 2.5 Western blot analysis

Total protein was extracted from cells using RIPA lysis buffer (Beyotime, China) supplemented with protease and phosphatase inhibitors (Roche, Switzerland). Protein concentrations were determined using the Bicinchoninic Acid Assay (BCA, Thermo Fisher, USA). Equal amounts of protein (30 μg) were separated by SDS-PAGE (sodium dodecyl sulfate polyacrylamide gel electrophoresis) and subsequently transferred onto PVDF (Polyvinylidene Difluoride) membranes (Millipore, USA). The membranes were blocked with 5% non-fat milk at room temperature and then incubated with primary antibodies at 4 °C overnight. The primary antibodies used included anti-ZNF486, anti-GATA2, anti-JAM2, anti-E-cadherin, anti-N-cadherin, anti-Vimentin, and anti-β-actin (all from commercial sources; catalog numbers and dilution ratios are provided in **Supplementary Table S3**). After washing, the membranes were incubated with Horseradish Peroxidase (HRP)-conjugated secondary antibodies at room temperature. Protein bands were visualized using an enhanced chemiluminescence (ECL) detection system (Bio-Rad, USA) and quantified by densitometry using ImageJ software (NIH, USA). β-actin served as the loading control for all Western blot experiments.

### 2.6 Immunofluorescence staining

Cells were seeded onto coverslips in 24-well plates and allowed to attach overnight. After attachment, cells were fixed with 4% paraformaldehyde for 20 min, permeabilized with 0.5% Triton X-100 for 10 min, and blocked with 5% bovine serum albumin (BSA) at room temperature for 1 h. The cells were then incubated with primary antibodies against E-cadherin (to assess membrane localization) and Vimentin (to evaluate cytoskeletal distribution) at 4 °C overnight. Following thorough washing, cells were incubated with Alexa Fluor 488- or 594-conjugated secondary antibodies (Thermo Fisher, USA) in the dark at room temperature for 1 h. Nuclei were counterstained with DAPI. Images were acquired using a confocal laser scanning microscope (Zeiss, Germany). Images were acquired using a 40× objective with identical laser power and gain settings across all experimental groups. For each condition, five random fields were captured, and over 100 cells were analyzed per group. E-cadherin membrane localization and Vimentin cytoskeletal distribution were assessed qualitatively.

### 2.7 Cell proliferation assay

Cell viability was assessed using the Cell Counting Kit-8 (CCK-8, Dojindo, Japan). Cells were seeded into 96-well plates at a density of 2 × 10³ cells per well, with five replicate wells per group. At 0, 24, 48, and 72 h after seeding, 10 μL of CCK-8 reagent was added to each well, and the plates were incubated at 37 °C for 2 h. The optical density (OD) at 450 nm was measured using a microplate reader (BioTek, USA).

### 2.8 Colony formation assay

Cells were seeded into 6-well plates at a density of 500–1,000 cells per well and cultured for 14 days. Once colonies were visible to the naked eye, the culture medium was discarded, and the cells were fixed with methanol and stained with 0.1% crystal violet. Colonies containing ≥50 cells were counted under an optical microscope. Each experiment was performed in triplicate wells and independently repeated three times. Colony counting was performed manually, and the investigator was blinded to the group allocation during both the counting and the selection of fields.

### 2.9 Transwell invasion assay

Cell invasion ability was evaluated using 24-well Transwell chambers with 8-μm pore size inserts (Corning, USA) pre-coated with Matrigel (BD Biosciences, USA, diluted 1:8). Cells were resuspended in serum-free medium and seeded into the upper chambers at a density of 1 × 10^5^ cells in 200 μL. The lower chambers were filled with 600 μL of culture medium containing 10% FBS as a chemoattractant. After 24 h of incubation, non-invading cells on the upper surface were removed with cotton swabs. Invading cells on the lower surface were fixed with methanol and stained with 0.1% crystal violet. Stained cells were counted in five randomly selected fields (200× magnification) under an optical microscope.

### 2.10 Wound healing assay

Cells were seeded into 6-well plates and cultured until 90–100% confluence. A straight scratch was created in the monolayer using a sterile 200-μL pipette tip. Debris was removed by washing with PBS (Phosphate-Buffered Saline), and the cells were cultured in serum-free medium. Images were captured at 0 h and 24 h using an inverted microscope (Olympus, Japan). The wound closure distance was measured using ImageJ software, and the healing rate was calculated as follows: (initial wound width − wound width at 24 h)/initial wound width × 100%.

### 2.11 Chromatin immunoprecipitation

ChIP assays were performed using a commercial ChIP kit (Millipore, USA) according to the manufacturer’s protocol. Cells were cross-linked with 1% formaldehyde at room temperature for 10 min, and chromatin was sheared by sonication to DNA fragments of 200–500 bp. Immunoprecipitation was carried out using anti-ZNF486 antibody, anti-GATA2 antibody, or normal rabbit IgG (as a negative control). After reversal of cross-linking, DNA was purified and subjected to qPCR with specific primers targeting the JAM2 promoter region (for ZNF486 ChIP) or the ZNF486 promoter region (for GATA2 ChIP). Enrichment was calculated as a percentage of input DNA. The data are presented as fold enrichment over the IgG control for clarity, which is a standard, albeit not definitive, method for initial analysis. ChIP-qPCR primer sequences are provided in **Supplementary Table S2**. We acknowledge that the inclusion of a negative control genomic region is a standard control that was not performed in this study, which represents a limitation of our ChIP-qPCR analysis.

### 2.12 Dual-luciferase reporter assay

Wild-type (WT) promoter fragments containing the predicted ZNF486 binding site (in the JAM2 promoter) or GATA2 binding site (in the ZNF486 promoter), as well as corresponding mutant (Mut) fragments, were cloned into the pGL3-Basic luciferase reporter vector (Promega, USA). Ishikawa and HEC-1A cells were co-transfected with WT or Mut reporter plasmids, the internal control plasmid pRL-TK (Promega, USA), and oe-ZNF486 (or oe-GATA2) or corresponding empty vector control using Lipofectamine 3000 (Invitrogen, USA). At 48 h post-transfection, firefly luciferase and Renilla luciferase activities were measured sequentially using the Dual-Luciferase Reporter Assay System (Promega, USA) on a GloMax^®^ Luminometer (Promega, USA). Firefly luciferase activity was normalized to Renilla luciferase activity to correct for transfection efficiency.

### 2.13 Statistical analysis

Data are shown as mean ± SD from three independent biological replicates (n=3), with three technical replicates per experiment. Comparisons between two groups were performed using two-tailed Student’s t-test, while comparisons among multiple groups were analyzed using one-way analysis of variance followed by Tukey’s *post hoc* test. All statistical analyses were conducted using GraphPad Prism 8.0 (GraphPad Software, USA). A P-value of < 0.05 was considered statistically significant (*P < 0.05, **P < 0.01, ***P < 0.001).

## 3 Results

### 3.1 ZNF486 is downregulated in UCEC and suppresses malignant phenotypes by inhibiting EMT

Analysis of the GEPIA2 database revealed that ZNF486 was significantly downregulated in UCEC (**Figure 1A**). Compared with normal endometrial epithelial cells (EM-E6/E7/TERT-1), ZNF486 mRNA and protein levels were significantly lower in both Ishikawa and HEC-1A UCEC cell lines (**Figures 1B, C**). Functional assays demonstrated that ZNF486 overexpression significantly upregulated E-cadherin, downregulated N-cadherin and Vimentin, enhanced E-cadherin membrane localization, and suppressed Vimentin cytoplasmic distribution (**Figures 1D–F**). Moreover, ZNF486 overexpression markedly inhibited cell proliferation, colony formation, migration, and invasion (**Figures 1G–K**). The overexpression efficiency, as shown in **Figure 1D**, resulted in an approximately 2-fold increase in ZNF486 protein levels compared to the oe-NC control, restoring expression to a level comparable to that seen in the normal EM-E6/E7/TERT-1 cells.

**Figure 1:**
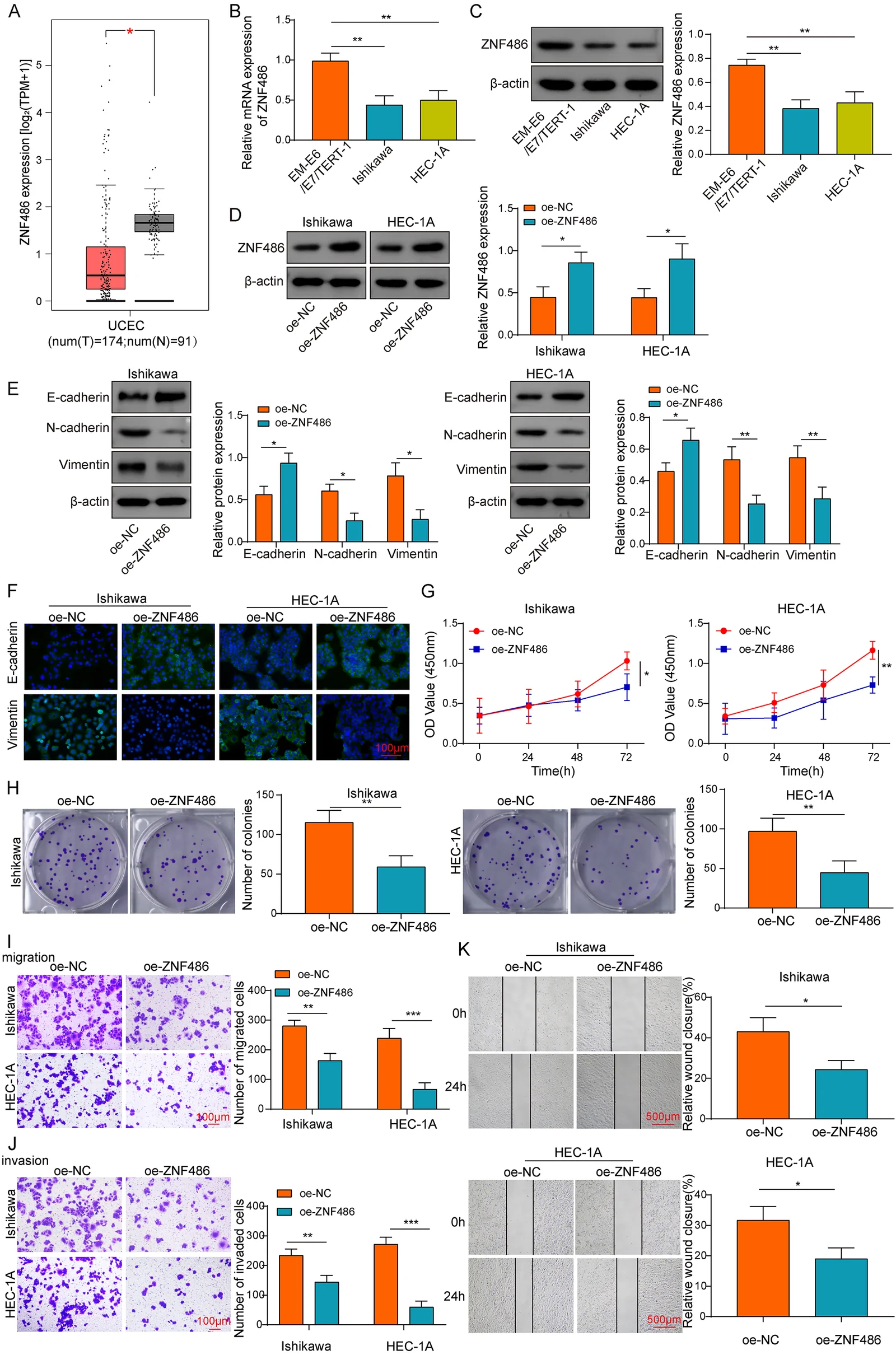
ZNF486 is downregulated in endometrial cancer and suppresses malignant phenotypes by inhibiting EMT. **(A)** Analysis of the GEPIA2 database (TCGA-UCEC dataset) showing significantly reduced ZNF486 mRNA expression in UCEC tissues compared with normal tissues. **(B, C)** qPCR **(B)** and Western blot **(C)** analyses of ZNF486 expression in normal endometrial epithelial cells (EM-E6/E7/TERT-1, Control) versus UCEC cell lines (Ishikawa and HEC-1A). **(D)** Verification of ZNF486 overexpression efficiency by Western blot in Ishikawa and HEC-1A cells infected with oe-ZNF486 or oe-NC control lentivirus. **(E)** Western blot analysis of EMT marker proteins (E-cadherin, N-cadherin, and Vimentin) following ZNF486 overexpression. **(F)** Immunofluorescence staining showing E-cadherin and Vimentin localization in oe-NC and oe-ZNF486 cells. Scale bar = 100 μm. **(G)** CCK-8 assays assessing cell proliferation at indicated time points. **(H)** Colony formation assays showing reduced colony numbers upon ZNF486 overexpression. **(I, J)** Transwell migration **(I)** and invasion **(J)** assays; representative images and quantitative data are shown. Scale bar = 100 μm. **(K)** Wound healing assays demonstrating impaired cell migration following ZNF486 overexpression. Scale bar = 500 μm. Data are presented as mean ± SD from three independent biological replicates. *P < 0.05, **P < 0.01, ***P < 0.001.

### 3.2 ZNF486 mediates its tumor-suppressive effects through direct transcriptional activation of JAM2

Database analysis showed that JAM2 was lowly expressed in UCEC and positively correlated with ZNF486 expression (**Figures 2A, B**). The JASPAR database predicted a potential binding motif for ZNF486 in the JAM2 promoter region (Motif ID: UN0815.1) (**Figure 2C**). JAM2 mRNA and protein levels were significantly lower in Ishikawa and HEC-1A cells than in EM-E6/E7/TERT-1 cells (**Figures 2D, E**). ChIP-qPCR and dual-luciferase reporter assays confirmed that ZNF486 shows significant occupancy at the JAM2 promoter and enhances its transcriptional activity (**Figures 2F–H**). Western blot (WB) confirmed successful JAM2 knockdown in sh-JAM2 cells (**Figure 2I**). Knockdown of JAM2 in ZNF486-overexpressing cells contributed to the ZNF486-mediated regulation of E-cadherin, N-cadherin, and Vimentin, and attenuated its inhibitory effects on proliferation, colony formation, migration, and invasion (**Figures 2J–P**). These findings indicate that ZNF486 exerts its tumor-suppressive functions, at least in part, through JAM2, prompting us to investigate the upstream regulators of ZNF486.

**Figure 2:**
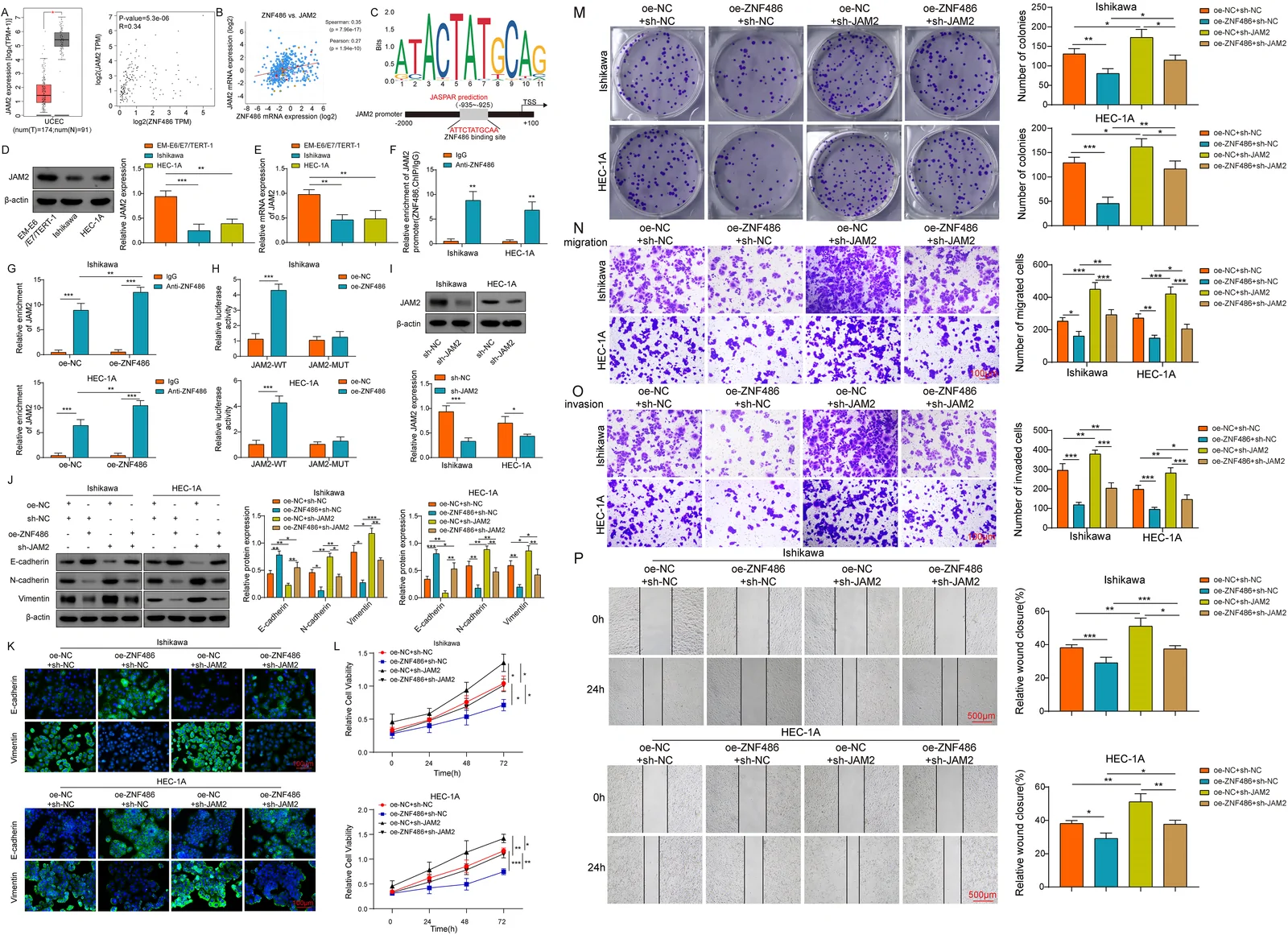
ZNF486 mediates its tumor-suppressive effects through direct transcriptional activation of JAM2. **(A)** GEPIA2 database analysis of JAM2 mRNA expression in UCEC versus normal tissues (left) and the correlation between JAM2 and ZNF486 expression in the TCGA-UCEC dataset (right). **(B)** cBioPortal analysis confirming a significant positive correlation between ZNF486 and JAM2 mRNA expression in UCEC (Spearman correlation coefficient and P-value are shown). **(C)** JASPAR-predicted ZNF486 binding motif (Motif ID: UN0815.1) in the JAM2 promoter region (motif logo) and schematic diagram of the putative ZNF486 binding site. **(D, E)** qPCR **(D)** and Western blot **(E)** analyses of JAM2 expression in normal endometrial epithelial cells (EM-E6/E7/TERT-1, Control) versus UCEC cell lines (Ishikawa and HEC-1A). **(F)** ChIP-qPCR assay evaluating ZNF486 occupancy at the JAM2 promoter in Ishikawa and HEC-1A cells. **(G)** ChIP-qPCR analysis showing increased ZNF486 enrichment at the JAM2 promoter upon ZNF486 overexpression (oe-ZNF486) compared with oe-NC control. **(H)** Dual-luciferase reporter assay demonstrating transcriptional activation of the JAM2 promoter by ZNF486 overexpression in Ishikawa and HEC-1A cells. **(I)** Western blot validation of JAM2 knockdown efficiency in cells transduced with sh-JAM2 versus sh-NC control. **(J)** Western blot analysis of E-cadherin, N-cadherin, and Vimentin expression in Ishikawa and HEC-1A cells subjected to the following treatments: oe-NC+sh-NC, oe-ZNF486+sh-NC, oe-NC+sh-JAM2, and oe-ZNF486+sh-JAM2. **(K)** Immunofluorescence staining of E-cadherin and Vimentin in the indicated treatment groups. Scale bar = 100 μm. **(L)** CCK-8 assays assessing cell proliferation at indicated time points. **(M)** Colony formation assays showing colony numbers in the indicated groups. **(N, O)** Transwell migration **(N)** and Matrigel-coated invasion **(O)** assays. Scale bar = 100 μm. **(P)** Wound healing assays showing cell migration at 0 and 24 h. Scale bar = 500 μm. Data are presented as mean ± SD from three independent biological replicates. the CCK-8 data were normalized to the respective 0-hour readings to account for any seeding differences. *P < 0.05, **P < 0.01, ***P < 0.001.

### 3.3 GATA2 directly activates ZNF486 transcription as an upstream transcription factor

To identify potential upstream regulators of ZNF486, we utilized the TF-Target Finder tool, which predicted several transcription factors (**Figure 3A**). Among these, we prioritized GATA2 for further investigation, as it has been established as a critical regulator of endometrial biology and its dysregulation has been frequently implicated in both benign and malignant endometrial disorders (19, 20). Database prediction and subsequent validation revealed that GATA2 is downregulated in UCEC and positively correlates with ZNF486 expression (**Figures 3A–E**). ChIP-qPCR and luciferase reporter assays demonstrated that GATA2 shows significant occupancy at the ZNF486 promoter and activates its transcription (**Figures 3F–K**). Overexpression of GATA2 significantly upregulated ZNF486 mRNA and protein levels, confirming GATA2 as an upstream transcriptional activator of ZNF486. Given these findings, we next sought to determine whether GATA2 suppresses UCEC malignant progression through the ZNF486-JAM2 axis.

**Figure 3:**
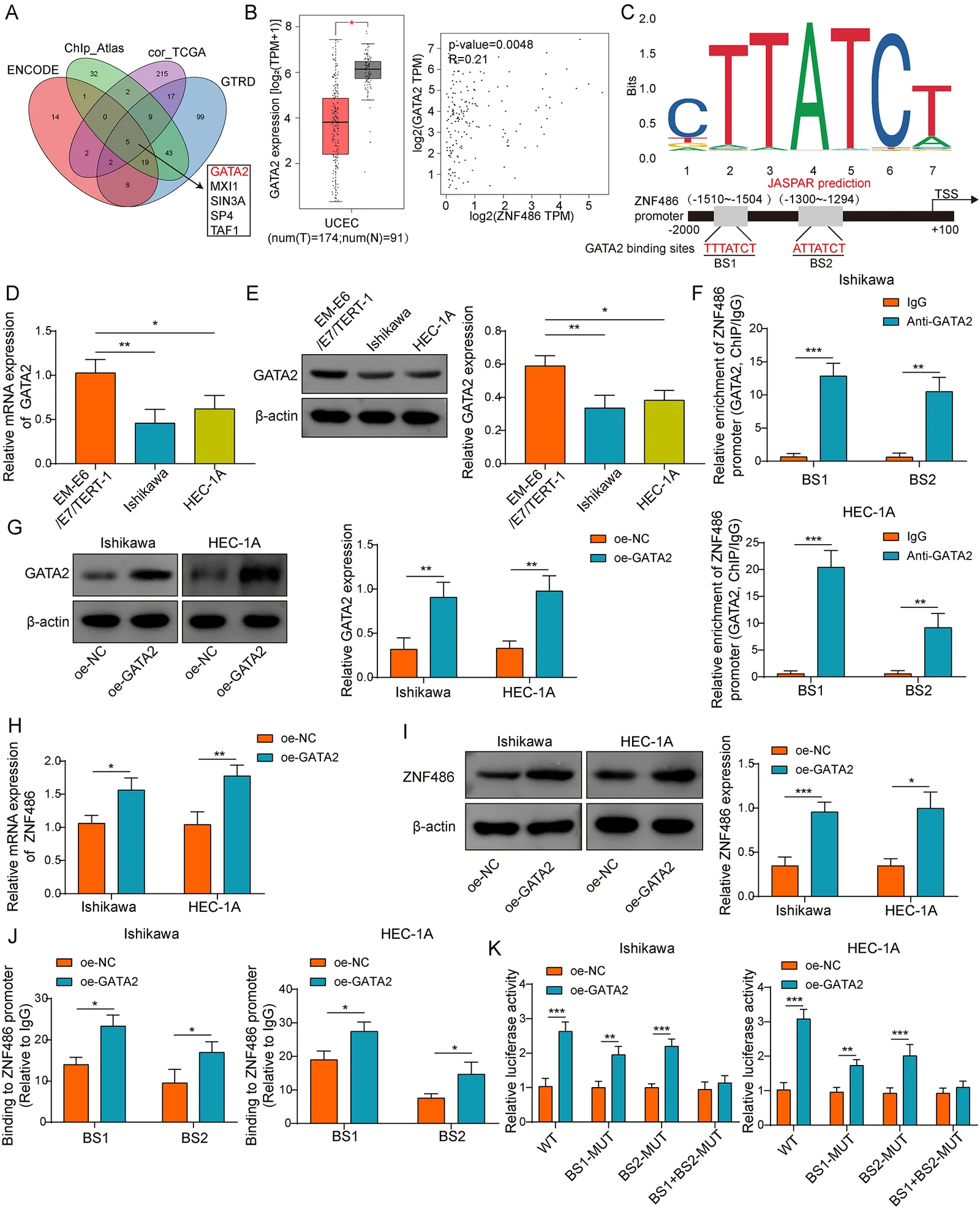
GATA2 directly activates ZNF486 transcription as an upstream transcription factor. **(A)** TF-Target Finder database prediction identifying GATA2 as a potential upstream transcriptional regulator of ZNF486. **(B)** GEPIA2 database analysis of GATA2 mRNA expression in UCEC versus normal tissues (left) and the correlation between GATA2 and ZNF486 expression in the TCGA-UCEC dataset (right). **(C)** JASPAR-predicted GATA2 binding motif (MA0036.4) in the ZNF486 promoter region (motif logo) and schematic diagram of the putative GATA2 binding site. **(D, E)** qPCR **(D)** and Western blot **(E)** analyses of GATA2 expression in normal endometrial epithelial cells (EM-E6/E7/TERT-1, Control) versus UCEC cell lines (Ishikawa and HEC-1A). **(F)** ChIP-qPCR assay evaluating endogenous GATA2 occupancy at the ZNF486 promoter in Ishikawa and HEC-1A cells. **(G)** Western blot confirmation of GATA2 overexpression efficiency in Ishikawa and HEC-1A cells transduced with oe-GATA2 or oe-NC control lentivirus. **(H, I)** qPCR **(H)** and Western blot **(I)** analyses of ZNF486 expression following GATA2 overexpression. **(J)** ChIP-qPCR analysis showing increased GATA2 enrichment at the ZNF486 promoter upon GATA2 overexpression (oe-GATA2) compared with oe-NC control. **(K)** Dual-luciferase reporter assay demonstrating transcriptional activation of the ZNF486 promoter by GATA2 overexpression in Ishikawa and HEC-1A cells. Data are presented as mean ± SD from three independent biological replicates. *P < 0.05, **P < 0.01, ***P < 0.001.

### 3.4 GATA2 suppresses UCEC malignant progression through the ZNF486-JAM2 axis

Knockdown of ZNF486 in GATA2-overexpressing cells significantly abrogated the GATA2-induced upregulation of ZNF486 and JAM2 (**Figures 4A–C**). Functional rescue experiments showed that ZNF486 knockdown partially attenuated the GATA2 overexpression-induced changes in EMT markers (upregulation of E-cadherin, downregulation of N-cadherin and Vimentin) and restored cell proliferation, colony formation, migration, and invasion (**Figures 4D–J**). In summary, GATA2 exerts its tumor-suppressive effects by activating the ZNF486-JAM2 axis, constituting a complete transcriptional regulatory pathway. It should be noted that while ZNF486 knockdown partially reduced ZNF486 and JAM2 expression, it did not fully abolish the GATA2-driven upregulation, consistent with the observed partial rescue of the phenotype.

**Figure 4:**
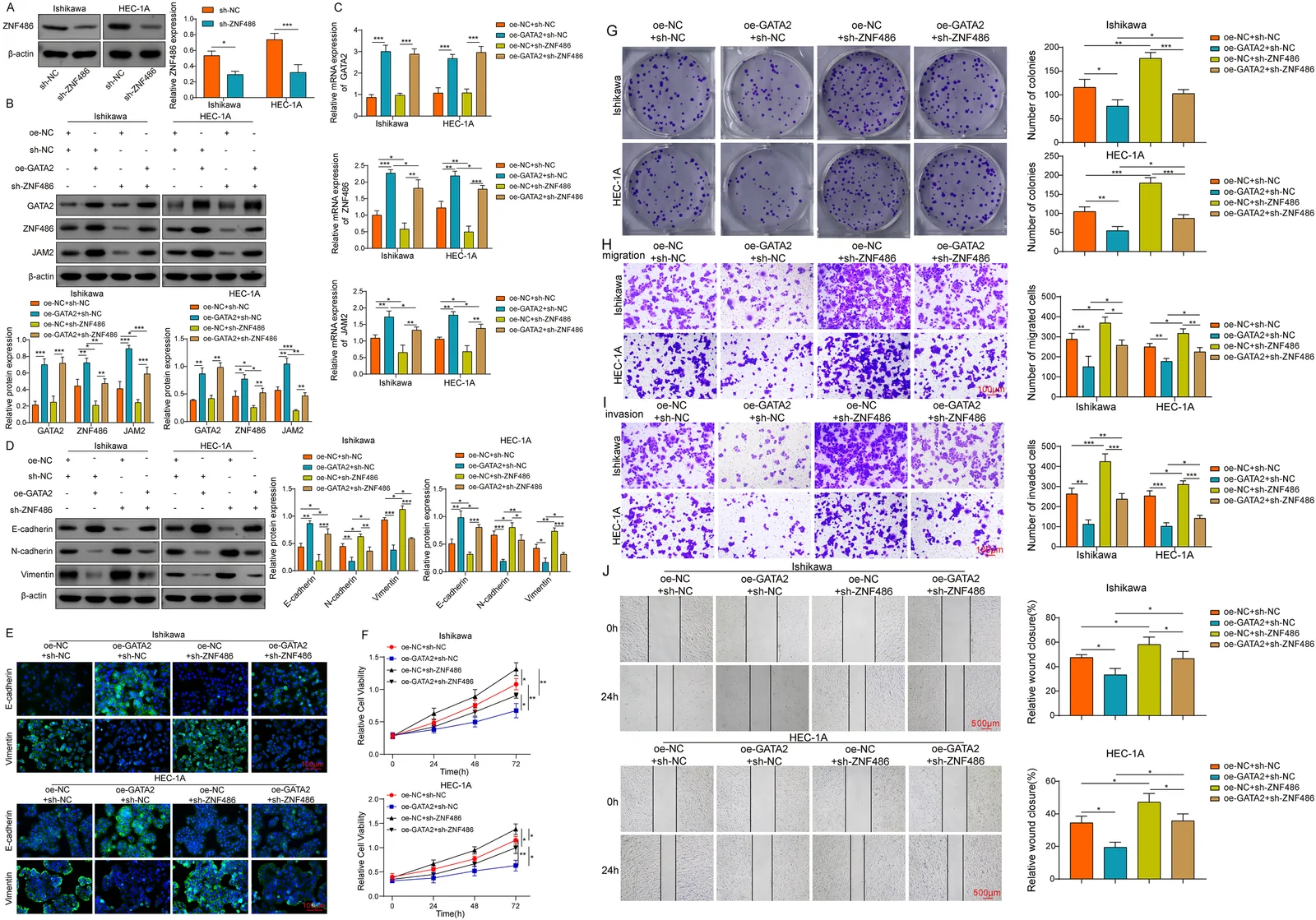
GATA2 suppresses UCEC malignant progression through the ZNF486-JAM2 axis. **(A)** Western blot validation of ZNF486 knockdown efficiency in Ishikawa and HEC-1A cells transduced with sh-ZNF486 or sh-NC control lentivirus. **(B, C)** qPCR **(B)** and Western blot **(C)** analyses of GATA2, ZNF486, and JAM2 expression in Ishikawa and HEC-1A cells subjected to the following treatments: oe-NC+sh-NC, oe-GATA2+sh-NC, oe-NC+sh-ZNF486, and oe-GATA2+sh-ZNF486. **(D)** Western blot analysis of E-cadherin, N-cadherin, and Vimentin protein levels in the indicated treatment groups. **(E)** Immunofluorescence staining of E-cadherin and Vimentin in the indicated treatment groups. Scale bar = 100 μm. **(F)** CCK-8 assays assessing cell proliferation at indicated time points. **(G)** Colony formation assays showing colony numbers in the indicated groups. **(H, I)** Transwell migration **(H)** and Matrigel-coated invasion **(I)** assays. Scale bar = 100 μm. **(J)** Wound healing assays showing cell migration at 0 and 24 h. Scale bar = 500 μm. Data are presented as mean ± SD from three independent biological replicates. *P < 0.05, **P < 0.01, ***P < 0.001.

## 4 Discussion

In this study, we identify the GATA2/ZNF486/JAM2 axis as a novel tumor-suppressive transcriptional cascade in UCEC. Through integrated *in vitro* and bioinformatics analyses, we demonstrate that this axis suppresses invasion and migration of UCEC cells, at least in part, by modulating EMT-associated marker expression. To our knowledge, this is the first report linking these three molecules into a single regulatory pathway in UCEC.

A key finding is the significant downregulation of ZNF486 in UCEC and its ability, upon overexpression, to enhance E-cadherin membrane localization and suppress Vimentin expression, suggesting a role as a guardian of the epithelial phenotype. This distinguishes ZNF486 from many other zinc finger proteins that predominantly promote EMT in cancer (21, 22). For instance, ZNF217 drives tumor progression in ovarian cancer (23), while ZNF281 and members of the ZIC and ZHX families exhibit opposing functions depending on tumor type (24–28). ZNF486 thus appears to occupy a unique functional niche within the zinc finger protein family.

Of particular note, ZNF486 contains a KRAB domain—a hallmark of transcriptional repressors—yet our data suggest it activates JAM2 transcription. Several non-mutually-exclusive explanations exist: (i) ZNF486 may indirectly activate JAM2 by repressing a transcriptional repressor; (ii) it may bypass its canonical repressive function through interactions with activator complexes or post-translational modifications; or (iii) the JASPAR motif (UN0815.1) used in our prediction, being from the “Unvalidated” collection, warrants caution. Future mechanistic studies are required to distinguish direct activation from indirect multi-step regulation.

Our previous observation of ZNF486 promoter methylation in UCEC (10) does not conflict with the present findings. Promoter hypermethylation and reduced transcription factor activity can co-occur to silence a tumor suppressor; indeed, methylation could preclude GATA2 binding, whereas in unmethylated cells, GATA2 levels may dictate transcriptional output. Such dual-layer regulation is common among tumor suppressors and merits further investigation.

Mechanistically, ChIP-qPCR and luciferase assays confirmed ZNF486 occupancy and transcriptional activation at the JAM2 promoter. The tumor-suppressive role of JAM2 has been well documented in lung adenocarcinoma, breast cancer, and colorectal cancer (13–15, 29), though it appears context-dependent—promoting progression in glioblastoma (30). Our rescue experiments indicated that JAM2 knockdown only partially contributed to the effects of ZNF486 overexpression, implying that ZNF486 may engage additional downstream effectors.

Upstream, we validated GATA2 as a transcriptional activator of ZNF486. GATA2 expression correlates positively with ZNF486 in UCEC, and GATA2 overexpression upregulates ZNF486 and JAM2, an effect blunted by ZNF486 knockdown. The role of GATA2 is highly tissue-specific: it acts as an oncogenic driver in prostate, pancreatic, and hepatocellular carcinomas (31–35), yet primarily exerts tumor-suppressive functions in endometrial and other hormone-related tumors (17, 19, 20, 36–39). Our study is the first to link GATA2, ZNF486, and JAM2 into a single signaling axis providing a novel downstream effector pathway for GATA2-mediated tumor suppression in UCEC. Future studies could investigate the protein expression patterns of GATA2, ZNF486, and JAM2 in clinical specimens using established resources such as The Human Protein Atlas to further validate the clinical relevance of this regulatory axis.

Several limitations should be acknowledged. All functional experiments were performed *in vitro*; *in vivo* validation is required. Our ChIP-qPCR data demonstrate promoter occupancy but lack a negative control locus, precluding a definitive claim of direct binding. Additionally, the use of a single shRNA per target gene raises the possibility of off-target effects; independent shRNAs or rescue constructs would strengthen specificity. Finally, we cannot exclude that ZNF486 modulates EMT through other signaling pathways (e.g., Wnt/β-catenin or PI3K/AKT).

In conclusion, this study reveals that the GATA2/ZNF486/JAM2 axis is inactivated in UCEC due to GATA2 downregulation, leading to reduced JAM2 expression and enhanced EMT-associated invasive and migratory capacity (**Figure 5**). These findings deepen our understanding of UCEC progression and provide a theoretical foundation for targeting this transcriptional axis.

**Figure 5:**
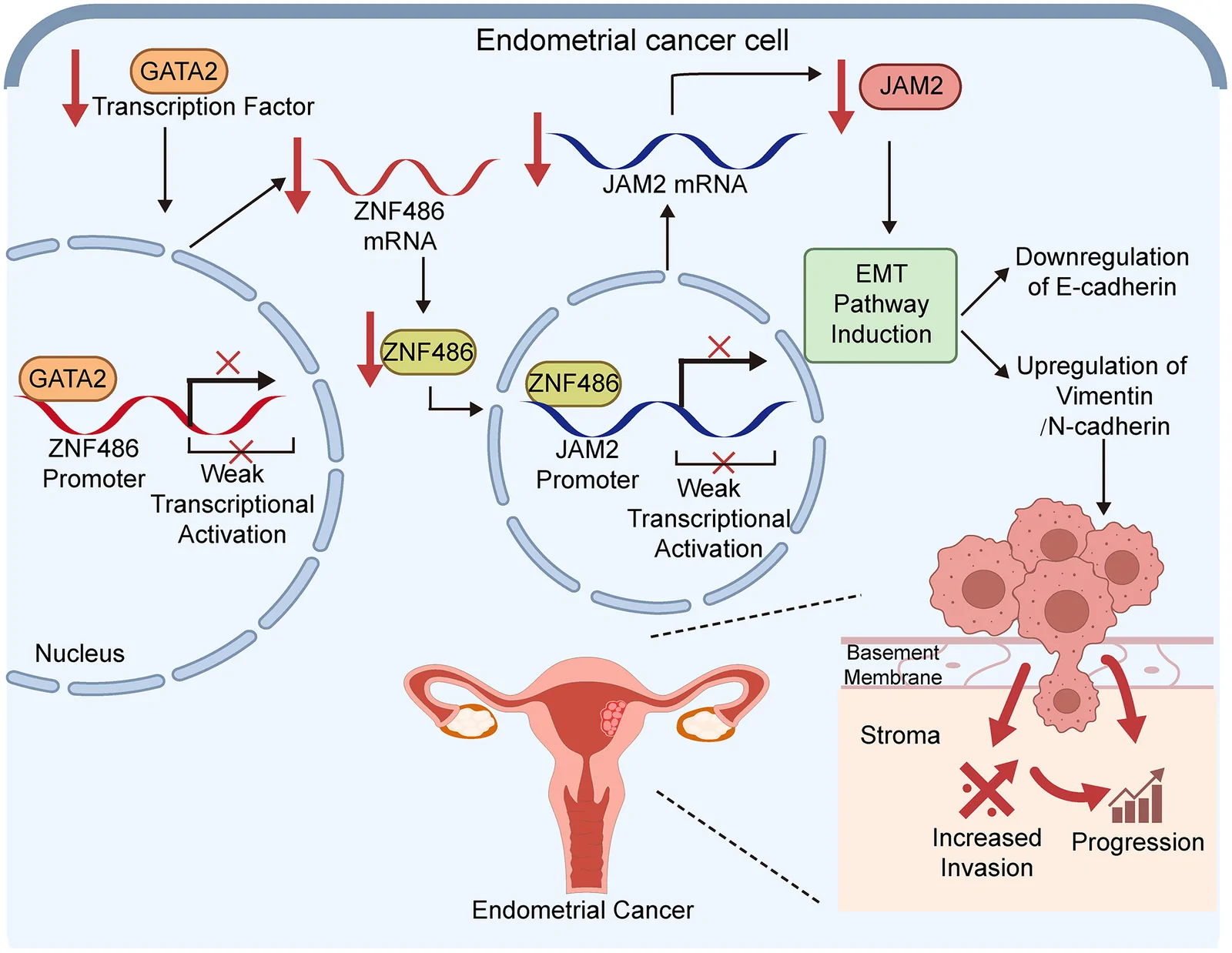
Schematic model of the GATA2/ZNF486/JAM2 transcriptional regulatory axis in endometrial cancer. In endometrial cancer, downregulation of the transcription factor GATA2 leads to reduced transcriptional activation of its downstream target gene ZNF486, resulting in decreased ZNF486 expression. The subsequent reduction in ZNF486 further diminishes its transcriptional activation of JAM2, causing JAM2 downregulation. Loss of JAM2 subsequently induces the expression of EMT-associated molecular markers, ultimately enhancing cancer cell invasion and driving disease progression. This figure summarizes the complete GATA2→ZNF486→JAM2 regulatory cascade and its functional consequences in UCEC.

## Data Availability

The raw data supporting the conclusions of this article will be made available by the authors, without undue reservation.
