# Supplementary material for: GATA2-driven ZNF486-JAM2 activation collaboratively counteracts epithelial-mesenchymal transition and in vitro invasion in endometrial cancer
Source: Front Oncol. 2026 Sep 15;16:1931641. doi: 10.3389/fonc.2026.1931641 (PMC13619468; doi:10.3389/fonc.2026.1931641)
Supplement: Supplementary file 1 [file Supplementaryfile1.docx]

Supplementary Table S1 cell lines

| Name | Source | Culture medium |
| --- | --- | --- |
| Ishikawa | Procell, Wuhan | DMEM/F12 +10%FBS-PS |
| HEC-1A | Procell, Wuhan |  |
| EM-E6/E7/TERT-1 | Borson, Shanghai |  |

Supplementary Table S2 Primer Sequences

| **Primers** | **F/R** | **Sequence (5’-3’)** |
| --- | --- | --- |
| GATA2 | F | GCAACCCCTACTATGCCAACC |
|  | R | CAGTGGCGTCTTGGAGAAG |
| ZNF486 | F | TGGACACTGCACAGCAGAAT |
|  | R | AACTGGGGGTTTGGCAATCA |
| JAM2 | F | GCAGTAGAGTACCAAGAGGCT |
|  | R | AGACACTCCGACCCAGTTTCT |
| ChIP-JAM2 | F | CCTTGGCTGGGTGGCAAATA |
|  | R | ACTCCAGTCAACCGAGGAGA |
| ChIP-ZNF486-BS1 | F | TTTCATCACTCAGCCCAGCA |
|  | R | TGATTGGACGGTTTCCAGCC |
| ChIP-ZNF486-BS2 | F | ATCCAATCAGAGACGCTGGG |
|  | R | GGAAGCCGTCCTCTTTGCT |

Supplementary Table S3 Antibodies

| **Antibody** | **Source** | **Item No.** | **Dilution ratio** |
| --- | --- | --- | --- |
| Anti-ZNF486 | Proteintech | 20599-1-AP | 1:500 |
| Anti-JAM2 | Abcam | ab156586 | 1:1000 |
| Anti-GATA2 | Proteintech | 11103-1-AP | 1:1500 |
| Anti-E-cadherin | Proteintech | 20874-1-AP | 1:20000 |
| Anti-N-cadherin | Proteintech | 22018-1-AP | 1:2000 |
| Anti-Vimentin | Proteintech | 10366-1-AP | 1:20000 |
| Anti-β-Actin | Cell Signaling Technology | 4970 | 1:1000 |
| Anti-rabbit IgG, HRP-linked Antibody | Cell Signaling Technology | 7074 | 1:1000 |
| Rabbit (DA1E) IgG Isotype Control | Cell Signaling Technology | 3423 | 1:20 |
| Mouse Anti-ZNF468 | Abcam | ab88862 | 10 µg/mL |
| Mouse IgG Isotype Control | Cell Signaling Technology | 3420 | 1:20 |
